# Hamilton’s Medical Jurisprudence

**Published:** 1888-01

**Authors:** 


**Hamilton's Medical Jurisprudence.** A Manual of Medical Jurisprudence, with special reference to Diseases and Injuries of the Nervous System. By ALLAN McLANE HAMILTON, M. D., one of the Consulting Physicians to the Insane Asylums of New York City, etc. Second edition; revised. New York: E. B. Treat, Publisher, 771 Broadway.

This is a practical work, doing away with those long and tiresome details which works on this subject so frequently give; yet all necessary details are given—plain, clear and concise. It is very fully illustrated with cases drawn largely from *American* sources, and, hence, better calculated to meet the wants of *American* physicians and legal advisers—a feature that is not always to be found in similar treatises. The leading chapters embrace Insanity in its Medico-legal Relations; Hysteroid Condition and Feigned Disease; Epilepsy; Alcoholism; Suicide; Cranial Injuries and Spinal Injuries. The first chapter defines Insanity, its general indications, classifications and hereditary influence — including Post-Mortem Examinations of the Insane (with plates of the typical and a typical brain). Under the Legal Relations of Insanity, we have Legal Tests—The Guiteau Case—Physical Tests—Duties of Medical Experts—Tricks of Counsel—Illusions, Hallucinations and Delusions—Reasoning Mania—Contracts made by the Insane—Testamentary Capacity—Old Age and Dementia —Undue Influence—Medico Legal Relations of Aphasia—Marriage and Insanity—Insurance Frauds—Responsibility of Deaf and Dumb—Criminal Responsibility—Responsibility in Relation to Imbecility—English Test of Responsibility; American Decisions on it—The Test of Right and Wrong—Impulsive Insanity — Commitment of Lunatics, and State Laws Regulating it—Concealed and Feigned Insanity, etc. The chapters on Cranial and Spinal Injuries are particularly valuable, for the numerous decisions cited from our courts in connection with suits for damages from railroad collisions, etc.

